# Fabrication of Anthocyanidin-Encapsulated Polyvinyl Alcohol Nanofibrous Membrane for Smart Packaging

**DOI:** 10.3390/nano14211701

**Published:** 2024-10-24

**Authors:** Maryam Aldoghaim, Jabrah Alkorbi, Salhah D. Al-Qahtani, Ghadah M. Al-Senani

**Affiliations:** 1 Department of Chemistry, College of Science, King Faisal University, Al-Ahsa 31982, Saudi Arabia; 2 Department of Chemistry, College of Science, The University of Sheffield, Sheffield S10 2TN, UK; jabrah.naji@gmail.com; 3 Department of Chemistry, College of Science, Princess Nourah bint Abdulrahman University, P.O. Box 84428, Riyadh 11671, Saudi Arabia; sdalohtany@pnu.edu.sa

**Keywords:** anthocyanin, cellulose nanocrystal-supported polyvinyl alcohol, electrospinning, nanofibrous membrane, smart packaging

## Abstract

Smart colorimetric packaging has been an important method to protect human health from external hazardous agents. However, the currently available colorimetric detectors use synthetic dye probes, which are costly, toxic, difficult to prepare, and non-biodegradable. Herein, an environmentally friendly cellulose nanocrystal (CNC)-supported polyvinyl alcohol (PVA) nanofibrous membrane was developed for the colorimetric monitoring of food spoilage. Anthocyanidin (ACY) is a naturally occurring spectroscopic probe that was isolated from pomegranate (*Punica granatum* L.). By encapsulating the anthocyanin probe in electrospun polyvinyl alcohol fibers in the presence of a mordant (M), M/ACY nanoparticles were generated. After exposure to rotten shrimp, an investigation on the colorimetric changes from purple to green for the smart nanofibrous fabric was conducted using the coloration parameters and absorbance spectra. In response to increasing the length of exposure to rotten shrimp, the absorption spectra of the anthocyanin-encapsulated nanofibrous membrane showed a wavelength blueshift from 580 nm to 412 nm. CNC displayed a diameter of 12–17 nm. The nanoparticle diameter of M/ACY was monitored in the range of 8–13 nm, and the nanofiber diameter was shown in the range of 70–135 nm. Slight changes in comfort properties were monitored after encapsulating M/ACY in the nanofibrous fabric.

## 1. Introduction

There has been a rising interest in the development of chromogenic detection tools toward smart packaging for food freshness monitoring. Therefore, consumers can easily distinguish between spoiled and fresh food, leading to improvements in food quality and human safety [1,2]. Several methods have been reported as potential detectors for food spoilage, such as amperometric and optical approaches [3,4]. However, those materials have shown high costs, long processing times, toxicity, irreversibility, maintenance hassles, and even high operating temperatures. Thus, it has been important to choose simple, environmentally friendly, and efficient advanced materials [5]. Solid-state colorimetric sensory materials have grown in popularity owing to their adaptability, affordability, and simplicity of application for real-time analysis. Smart textiles change color in reaction to many environmental conditions, such as heat and light [6,7,8,9].

The improved permeability to liquid and gaseous analytes, reduced weight, and large surface area differentiates nanofibrous membranes from dense materials [10]. Polyvinyl alcohol (PVA) is a hydrophilic polymer prepared by a hydrolysis reaction of polyvinyl acetate, which is generated by the polymerization of vinyl acetate [11]. Due to its nontoxicity, biocompatibility, biodegradability, and wide availability, PVA has been used in various fields, such as tissue engineering [12]. PVA nanofibrous fabrics have shown several potential applications in both medical and environmental sciences. PVA-based nanofibrous composite membranes have been known to easily produce combinations with a number of active chemical agents [13,14,15]. The aforementioned advantages of PVA-based composite membranes are ascribed to their nanofibrous architecture with a large surface area [16]. The higher sensitivity to gas and liquid analytes is a consequence of the membrane nanostructure, which causes rapid matrix diffusion and high surface adsorption [17]. There are a number of techniques that have been presented for detecting rotten food, such as electrochemical and chromatography tools [18,19]. However, these detection processes have shown to be costly, unselective, nonportable, and slow in terms of sensitivity. Additionally, they necessitate trained staff and complicated electronic and/or electrical parts. In order to provide a real-time detection, food spoilage detectors need to be responsive in a sub-second timeframe [20]. Nanomaterials imprinted with active probes have been efficiently used to detect a certain analyte via fluorescent and/or colorimetric methods [21,22]. There have been many efficient approaches reported for the fluorescence identification of primary hazards. However, fluorescent detectors have shown disadvantages, such as paramagnetic-induced fluorescence quenching and low solubility in aqueous solutions [23]. Additionally, the preparation of fluorescence sensors requires costly and time-consuming purification [24]. On the other hand, colorimetric detectors have shown many advantages, such as simple miniaturization, low operational costs, and independence from batteries and wires. Additionally, colorimetric sensors require no specialist staff [25]. Thus, various colorimetric sensors have been reported using synthetic sensor probes, such as hydrazones and boron dipyrromethenes, for detection of various analytes by incorporating an appropriate sensor dye into a hosting substrate with a large surface area [26,27]. However, it has been a challenge to develop a standard colorimetric detector for food spoilage owing to the lack of sensor dyestuffs with desired characteristics, such as biocompatibility, high sensitivity, biodegradability, and simple processing [28]. Anthocyanins are a group of pH-responsive dyes found naturally in many common plants, such as red cabbage and pomegranate. Due to its low price, robust growth output, and widespread availability, the pomegranate plant has been a valuable anthocyanin resource [29,30]. The colorimetric transitions shown by anthocyanins in aqueous solutions range between colorless in highly basic solutions, red at extremely acidic pH values, purplish at neutral pH 7, greenish at slightly basic pH values, and pink at slightly acidic pH values. The fact that anthocyanins are present in the tissues of numerous food plants means that they are not toxic. They have been used as food colorants. They have also shown antioxidant and anticancer activity [31,32,33].

Cellulose nanocrystals (CNCs) have been used as a reinforcement agent for various polymer composites for the preparation of mechanically reliable biomaterials [34,35,36,37,38]. CNCs have been used in nanomedical applications, such as biosensors, antimicrobial agents, tissue substituents, and bioimaging agents. CNCs have received significant interest due to their chemical, optical, and mechanical properties [34,35]. CNCs can be synthesized from a variety of raw materials, such as cotton, pulp, wood, and straw. Some of these resources require pretreatment to remove hemicellulose and lignin, providing cellulose of high purity to be used for the synthesis of CNCs. CNCs have been successfully prepared using ultrasound treatment and acid hydrolysis. This ultrasound-assisted acid hydrolysis procedure has proven an efficient approach for the synthesis of CNCs [36,37,38]. Microcrystalline cellulose has been used as a significant source of CNCs. CNCs have been characterized as having a large surface area, a reactive surface, lightweight biodegradability, and low cost [39]. However, the hygroscopic nature of CNCs has shown disadvantages, such as weak adhesion to nonpolar polymer agents and excessive adsorption of moisture. Therefore, the chemical structure of CNCs has been modified to produce important functional substituents able to control the phase of additive bonding [40].

Herein, the colorimetric monitoring of rotten shrimp was achieved by using an anthocyanin-encapsulated cellulose nanocrystal-reinforced polyvinyl alcohol nanofibrous membrane. The present anthocyanin-encapsulated cellulose nanocrystal-reinforced polyvinyl alcohol nanofibrous sensor is simple, easy to use, very sensitive, portable, reversible, and cheap. There is no need for specialized personnel, complicated tools, or electrical wiring. It was prepared by using a mordant (aluminum salt) to fasten anthocyanin onto CNC@PVA nanofibers. The current sensor displayed a colorimetric change from purplish to greenish in response to the total volatile basic nitrogenous amines (TVB-Ns), such as ammonia, and nitrogen-containing amines, released from food spoilage. The large surface area of the CNC@PVA nanofibrous membrane results in the strong adsorption capacity of the generated M/ACY particles, leading to the accurate monitoring of food spoilage [41]. TVB-Ns released from food spoilage causes colorimetric changes due to charge delocalization on anthocyanin molecules caused by a reversible protonation and deprotonation process. The colorimetric studies were conducted using the colorimetric coordinates and absorption spectral analysis. Energy-dispersive X-ray (EDX), scanning electron microscopy (SEM), and transmission electron microscopy (TEM) were utilized to inspect the sensor morphology. In order to ensure the safety of consumers, the present study incorporates an *onsite* detection system that can identify the total volatile basic nitrogenous amines (TVB-Ns) released from food spoilage in various products.

## 2. Experimental Design

### 2.1. Materials

The PVA (99%; Mw 89,000–98,000 g/mol), maleic anhydride (MA; 99%), hydrochloric acid (ACS reagent, 37%), microcrystalline cellulose (MCC; particle size of 20 μm), sodium chloride (ACS reagent, 99.0%), absolute ethanol (95.0%), and potassium aluminum sulfate (potash alum; KAl(SO_4_)_2_·12H_2_O) were procured from Merck (Darmstadt, Germany). The magnesium sulfate (MgSO_4_), hydrogen peroxide (H_2_O_2_), sodium hydroxide (NaOH), sodium silicate, and diethylene triamine pentaacetic acid (DTPA) were supplied by Aldrich (Darmstadt, Germany). The anthocyanidin extract was obtained from the pomegranate plant (*Punica granatum* L.; Saudi Arabian farms) by using a formerly reported method with some modifications [29]. The MA-modified CNC was prepared using a previous procedure [42].

### 2.2. Preparation of MA-Modified CNC

Using sulfuric acid (100 mL; 65%), MCC (10 g) was exposed to acid hydrolysis in a water bath at 45 °C by a mechanical stirring for 2.5 h. Then, the hydrolysis reaction was stopped by adding ultrapure water (900 mL). After centrifuging the precipitate-generating mixture at 8000 rpm numerous times, an opaque blue layer was observed. After a dialyzation with ultrapure water for 7 days by using a dialysis membrane with a cut-off of 12,000 Da to 14,000 Da (Fisherbrand, Pittsburgh, PA, USA), the supplied liquid was homogenized in an ice bath for 45 min to generate CNCs. MA (8 g) was allowed to completely melt at 130 °C. The resulting melt was then admixed with the CNCs (1 g) and stirred for 20 h. After adding 20 mL of dimethylacetamide, the combination was stirred for 30 min. Following vacuum filtration, the combination was rinsed with distilled water, and then dried at 125 °C for 21 h.

### 2.3. Anthocyanin Extraction

Finely chopped pomegranate (300 g) was scoured for 45 min with NaCl_(s)_ (30 g). Then, the combination was subjected to maceration in distilled water (500 mL) for 10 h. After filtering and centrifuging for 20 min at 2200 rpm, a purple solution with a pH of ~6.7 was produced. The produced extract was treated with HCl_(aq)_ and placed in a freezer at 3 °C for 3 days. The generated precipitate was isolated by filtration under vacuum and air-dried for 15 h. The produced anthocyanin was reserved in a fridge at 5 °C for additional examination. An Agilent 1100 (Waldbronn, Germany) was utilized to analyze the extract according to the previously reported procedure [29,30,31]. Additionally, a Shinoda experiment was performed on the extracted solution (5 mL) to produce a red color when combined with a piece of Mg ribbon and HCl (1 mL) [29,30,31,32,33].

### 2.4. Preparation of Chromic Membranes (ACY/CNC@PVA)

**Scheme 1:**
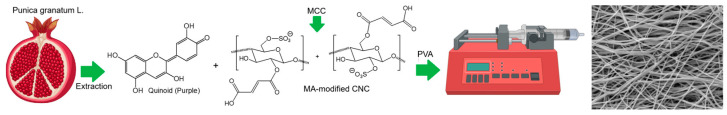
Preparation procedure of chromic membrane (*ACY*/CNC@PVA).

A homogeneous solution was produced by dissolving PVA (14% *w*/*v*) in distilled water, and homogenizing the provided mixture at 35 kHz for 20 min. The provided solution was mordanted with potash alum (5% *w*/*v*), admixed with CNCs (5% *w*/*w*), and then stirred for 30 min, and homogenized at 35 kHz for 30 min. The pH of the viscous composite was brought down to 6.5 by using acetic acid. The solid extract of pomegranate was then added at different ratios to the produced viscous solution (CNC@PVA), including 0% (ACY_0_), 0.25% (ACY_1_), 0.5% (ACY_2_), 0.75% (ACY_3_), 1% (ACY_4_), 1.25% (ACY_5_), 1.5% (ACY_6_), and 1.75% (ACY_7_). Using the electrospinning technology, a needle tip (stainless steel; ~1.30 mm) connected to a programmable plastic syringe was held 22 cm away from the collector (aluminum foil; 20 × 20 cm). The electrospinning process was carried out at a high voltage of 15 kV and a flow rate of 0.7 mL/h in a wooden ventilated fume cupboard at room temperature. The nanofibrous membranes were then entrapped between two glass slides and dried at 40 °C. Scheme 1 displays the preparation procedure for the chromic membrane.

### 2.5. Analysis Methods

The morphologies of the ACY-encapsulated membranes were inspected using VEGA3 TESCAN (TESCAN, Brno, Czech Republic) at 25 kV. A sputtering instrument (BIO-RAD Unit PS3) was used to coat the sample with a 5 nm thick gold film for SEM analysis. The elemental compositions of nanofibers were investigated by TEAM-EDX. By using a Nicolet Nexus-670 FTIR spectrometer (Thermo Fisher, Waltham, MA, USA) and the Attenuated Total Reflection (ATR) mode, the substituents on the ACY-encapsulated nanofibers were investigated. The diameter and shape of the M/ACY nanoparticles were determined by JEOL-1230 transmission electron microscopy (Tokyo, Japan). After washing with distilled water, the colored nanofibers (ACY_5_) were centrifuged. The nanofibrous membrane was removed, and the generated aqueous suspension was homogenized for 60 min. Drops of the M/ACY nanoparticle-containing aqueous suspension were then decanted on a copper grid for TEM analysis. In order to measure the stiffness of the nanofibrous membranes, the Shirley stiffness tool (British standard 3356:1961) was used [43]. The air permeability was measured by a FX-3300 according to the ASTM D737 procedure [44]. Using Quantachrome Touch-Win software at room temperature, the membrane surface area was measured by an N_2_ adsorption–desorption isotherm [45].

### 2.6. Colorimetric Measurements

The spoilage monitoring was tested by the direct contact of the optimum cellulose nanocrystal-reinforced polyvinyl alcohol nanofibrous membrane (ACY_5_) with shrimps. A colorimetric change was monitored from purple to green over 6 to 30 h. Using an UltraScanPro (Hunter Lab, Reston, VA, USA), the absorption spectra, the CIE Lab dimensions, and the color strength (*K/S*) were then determined [46]. Photographs of ACY_5_ were shot during the exposure time to shrimps using a Nikon D850. The sensor reversibility (ACY_5_) was tested by using shrimp spoilage to create a green color. After exposure to air for 1 h, the green nanofibrous membrane reverted back to its purple hue. A HunterLab Ultrascan Pro (USA) was utilized to report the absorbance spectra after numerous cycles of exposure to shrimps for 30 h, rinsed with distilled water, and air-dried. The colorimetric properties of the ACY-encapsulated CNC@PVA nanofibers were studied using an Ultrascan Pro spectrophotometer. For the CIE Lab coordinates, L* is the lightness coordinate between white (100) and black (0), the a* coordinate ranges between greenish (−) and reddish (+), and the b* coordinate ranges between blue (−) and yellow (+) [47]. The pH value was determined by using an ADWA AD-11 pH meter. Standard ISO105 procedures, such as B02:1988, C02:1989, and E04:1989, were used to determine the durability of the developed membranes to light, washing, and perspiration, respectively [48].

### 2.7. Statistical Analysis

The statistical studies were reported using Prism-GraphPad software (version 6.0, San Diego, CA, USA). The experimental procedures were replicated three times to approve the repeatability of the reported findings. The findings were recorded as the average ± standard deviation (SD). The statistical significance was determined by using a one-way *ANOVA* as the *p*-values were calculated to evaluate the difference between groups.

## 3. Results and Discussion

### 3.1. Preparation of Chromic Membranes

Anthocyanins generate a colorimetric change when exposed to food spoilage. Anthocyanins have been recognized as a halochromic class of naturally occurring dyestuffs. When altering the pH value, anthocyanins produce different colors, including (strongly basic) colorless, greenish yellow (mildly basic), purple (neutral), pink (mildly acidic), and red (severely acidic) [32]. These colorimetric changes are assigned to charge delocalization driven by the molecular switching of anthocyanin. Due to their small molecular size, non-toxicity, high sensitivity, quick monitoring, biodegradability, biocompatibility, and water-solubility, anthocyanins have proved effective indicators for food spoilage. Anthocyanin was extracted from chopped pomegranate by scouring with NaCl_(s)_ and macerating in distilled water, producing a purple extract. The Shinoda experiment was employed to verify the anthocyanin flavonoid characteristic to produce a red hue [29,30,31,32,33], thereby confirming the presence of anthocyanidin. Additionally, HPLC was used to analyze an extract solution in methanol. The existence of bioactive phenols was verified by HPLC; however, the overall amounts of these phenols varied, as illustrated in Table 1. Cellulose nanocrystal-reinforced polyvinyl alcohol nanofibrous membranes were dyed in situ during the electrospinning process. As a result of their water solubility, the colorfastness of anthocyanins is severely compromised when washed several times. Thus, a viscous solution of CNC-reinforced PVA was amended with potash alum to strongly attach anthocyanin into the CNC-reinforced PVA nanofibrous fabric. In this halochromic detection experiment, a colorimetric change was observed from purple to green after exposure to rotten shrimp.

### 3.2. Morphological Characterization

**Figure 1:**
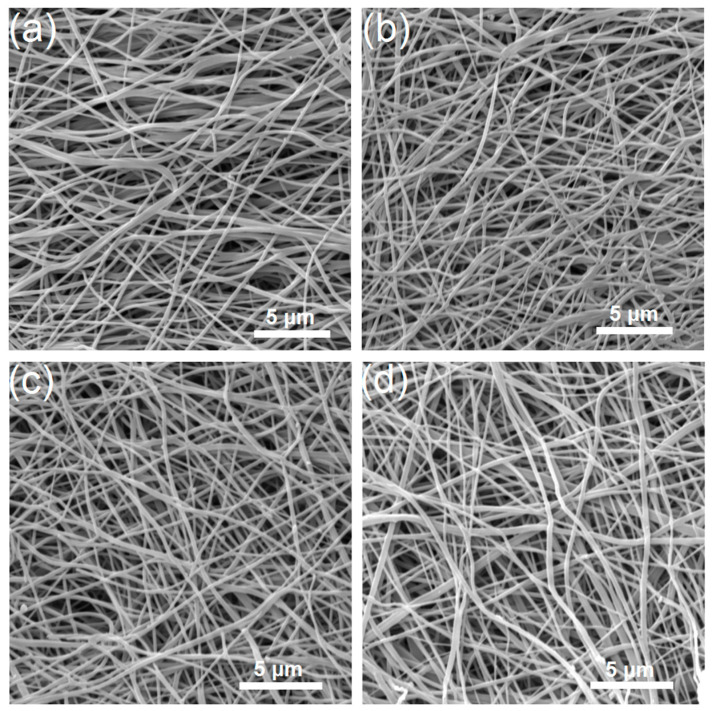
SEM images of ACY_5_ at different locations on the sample surface (**a**–**d**), indicating a homogeneous surface.

Chromogenic sensors are distinguished by their portability, quick detection, ease of use, reversibility, and low cost. Thus, the development of reliable colorimetric sensors for the determination of analytes has been a significant demand. Furthermore, the search for environmentally friendly colorimetric sensors has been critical [49]. Polyvinyl alcohol nanofibrous membranes have been important materials for various uses owing to their porous structure, light weight, and large surface area. Various methods were used to examine the ACY-encapsulated nanofibrous membranes. As a result of their water solubility, most natural dyestuffs have shown a weak affinity for hydrophobic fabrics. Thus, a mordant must be used to improve their fastness properties by the formation of water-insoluble M/ACY particles [41]. The M/ACY nanoparticles were generated by applying anthocyanin to the fibrous membrane in the presence of potash alum. The M/ACY nanoparticles were formed in situ in the produced nanofibers during the electrospinning process. Figure 1 displays SEM images of the ACY/CNC@PVA detector. The developed nanofibers exhibited diameters of 70 to 135 nm. TEM images demonstrated particle diameters of M/ACY in the range of 8–13 nm. Thus, the high sensitivity of the M/ACY nanoparticle sensor layer on the cellulose nanocrystal-reinforced polyvinyl alcohol nanofibrous membrane to trace analytes is a consequence of the membrane nanostructure, which causes a high surface adsorption and a quick diffusion for the membrane matrix [30]. As a result of its large surface area, the halochromic sensor has a better sensitivity to food spoilage. After encapsulating M/ACY nanoparticles into the bulk of CNC@PVA, the membrane porosity remained unchanged [30]. Thus, the nanofibrous matrix was able to facilitate the adsorbed and diffusion of TVB-N to the M/ACY nanoparticles as active detection sites. The surface area of a fibrous sample (ACY_5_) was reported at 29 ± 1.1 m^2^/g. Gouda et al. reported recently the development of an anthocyanin-encapsulated cellulose acetate nanofibrous membrane, demonstrating almost a similar surface area of 26 m^2^/g [30]. The high sensitivity of the sensor to food spoiling is ascribed to its high surface area. The adsorption of TVB-Ns generated by spoiled food onto the membrane surface and their diffusion through this membrane matrix into the M/ACY nanoparticles were facilitated by the membrane’s large surface area. The elemental contents of both blank and ACY-encapsulated fabrics at three separate sites are summarized in Table 2. It was found that the chemical compositions at the three scanned sites were almost comparable, assuring a consistent and uniform distribution of M/ACY nanoparticles on the membrane surface.

**Figure 2:**
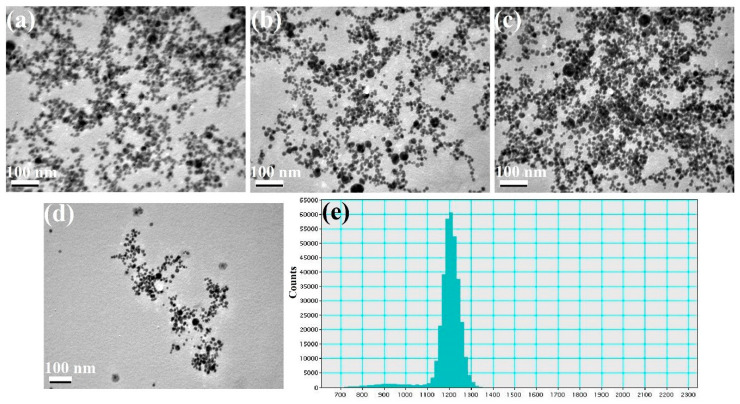
TEM images of M/ACY nanoparticles at different positions of the sample (**a**–**d**), indicating homogeneous diameters of nanoparticles and particle size distribution of M/ACY nanoparticles (**e**).

**Scheme 2:**
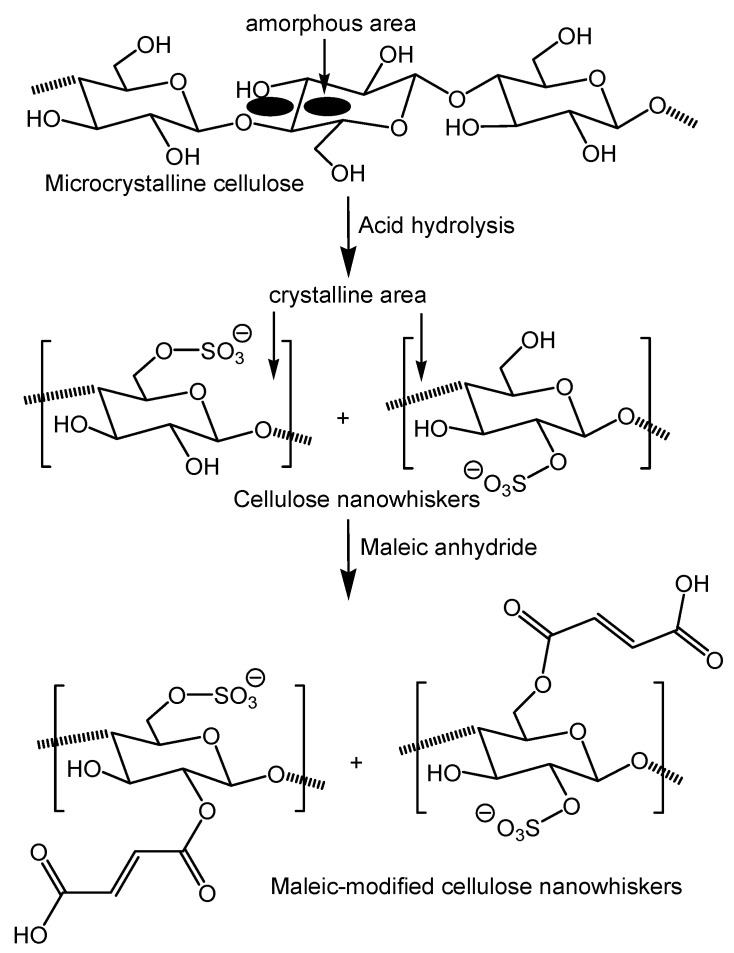
Preparation of maleic-modified CNC.

**Figure 3:**
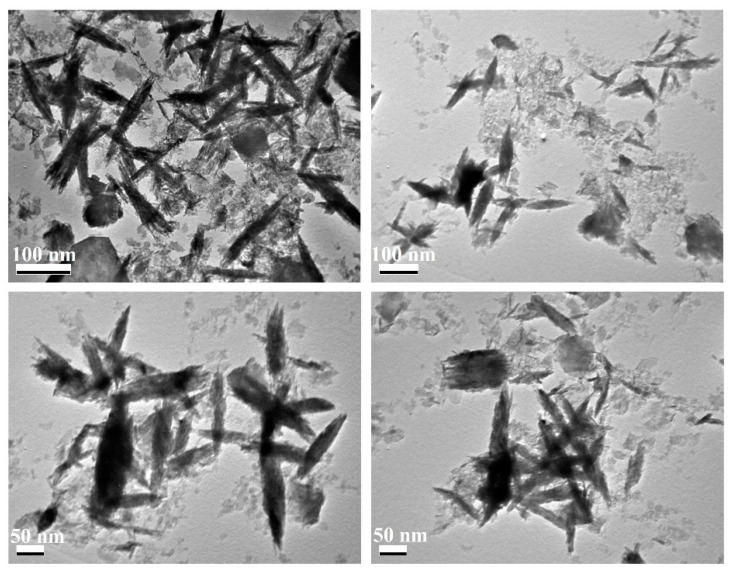
TEM analysis of the CNC at different positions and different magnifications of the sample.

Transmission electron microscope (TEM) micrographs of M/ACY nanoparticles show diameters of 8–13 nm (Figure 2a–d). Figure 2e shows the particle size distribution of the M/ACY nanoparticles, proving a homogeneous particle diameter. The colored membrane (ACY_5_) was rinsed with distilled water and homogenized to isolate M/ACY nanoparticles. The aqueous suspension was then centrifuged and homogenized for TEM examination. The CNC was prepared from microcrystalline cellulose, as shown in Scheme 2 [42]. TEM analysis of the CNC showed a diameter of 12–17 nm, as illustrated in Figure 3. The CNC was used to reinforce PVA to enhance its mechanical strength as the CNC-free PVA nanofibrous membrane dissolves in water. Additionally, the CNC was used as a dispersant to prevent aggregation of M/ACY nanoparticles.

The substituents on the fibrous samples were determined by FTIR analysis (Figures S1–S3). Both hydroxyl and aliphatic peaks were determined at 3354 cm^−1^, and 2932 cm^−1^, respectively. No changes were detected in the FTIR spectra of the nanofibrous membrane before and after exposure to rotten shrimp. The deformation of water molecules displayed a vibration band at 1617 cm^−1^. When the ACY content was increased, the hydroxyl absorption band was found to slightly decrease due to the increased coordination bonds between aluminum, anthocyanin, and PVA hydroxyl [29,30].

### 3.3. Comfort and Fastness

**Figure 4:**
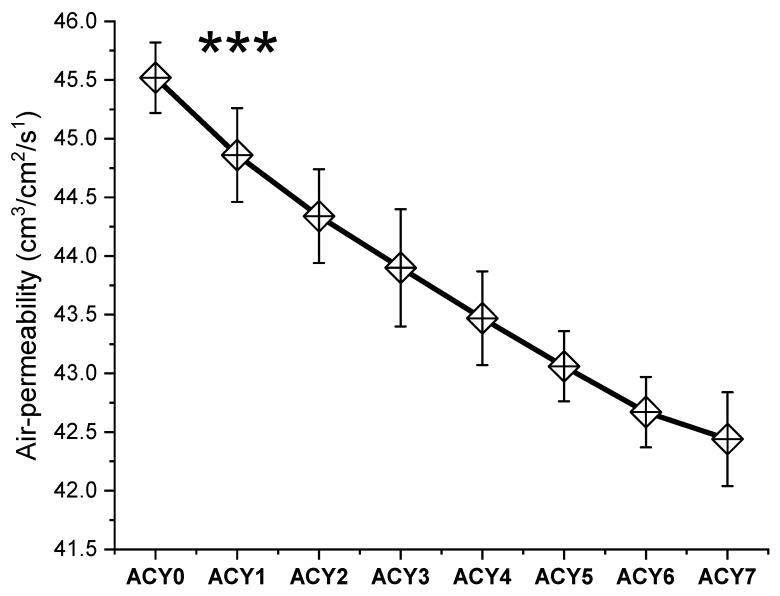
The air permeability of membranes. Using the one-way *ANOVA*, the relationship levels are defined as *** high (*p* ≤ 0.001).

**Figure 5:**
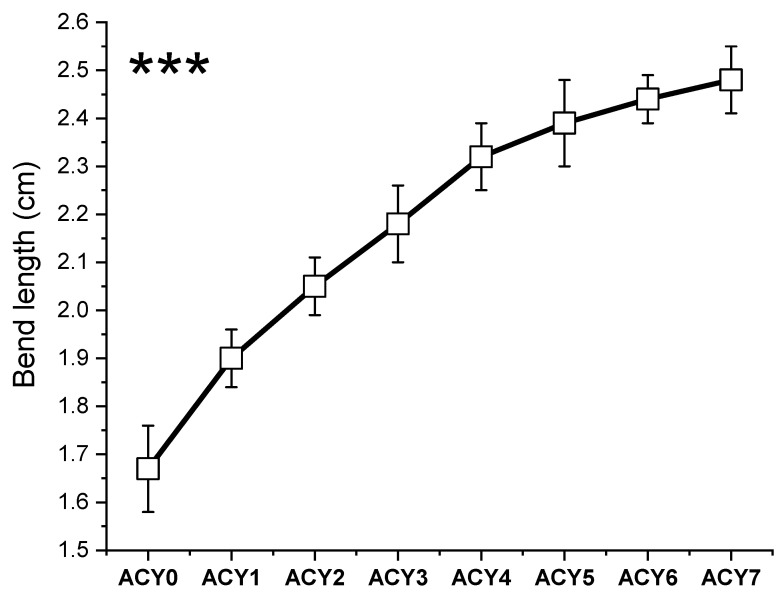
The bending lengths of membranes. Using the one-way *ANOVA*, the relationship levels are defined as *** high (*p* ≤ 0.001).

The primary purpose of the present study is to develop a chromic textile that is both highly breathable and very flexible. The coloration course did not alter the air permeability of membranes (Figure 4); nonetheless, increasing the anthocyanin concentration resulted in a small increase in stiffness (Figure 5). The nanofibrous membranes showed good durability and colorfastness against washing, light, and perspiration, as illustrated in Table 3. Nonetheless, the fastness against light slightly decreased with increasing the anthocyanin content.

### 3.4. Colorimetric Screening

**Figure 6:**
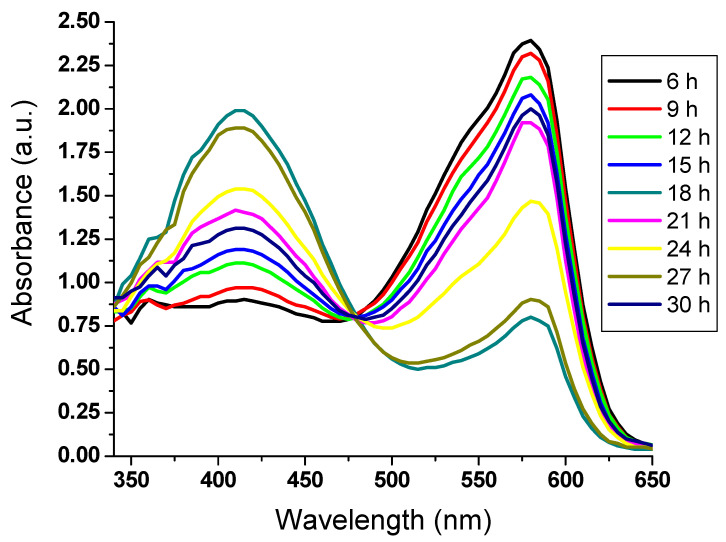
Absorbance spectra of ACY_5_ against the exposure period to rotten shrimp (6–30 h) demonstrating a wavelength shift from 580 nm to 412 nm.

The sensor efficiency was influenced by the ACY ratio on the membrane surface. Table 4 illustrates the coloration measurements of the CNC-reinforced polyvinyl alcohol nanofibrous membranes encapsulated with varying amounts of ACY. The anthocyanin-free membrane (ACY_0_) demonstrated no considerable alteration in its colorimetric properties after exposure to food spoilage (30 h). After exposure to food spoilage (30 h), the maximum absorption wavelength of the anthocyanin-containing membranes changes hypsochromically from 580 nm to 412 nm (Figure 6). As the anthocyanin extract was increased, the color strength of the membranes was improved from ACY_1_ to ACY_5_. However, a slight enhancement was observed in *K/S* with an increasing ACY ratio from ACY_5_ to ACY_7_. Thus, ACY_5_ can be described as the best sample due to its optimum colorimetric activity. Additionally, the color strength of the nanofibrous membranes was highly decreased after exposure to food spoilage (30 h) due to a change in color from purplish to green. The CIE Lab parameters of the anthocyanin-containing membranes were considerably different from those of ACY_0_. Increasing the anthocyanin content from ACY_1_ to ACY_5_ resulted in decreasing the value of L* to indicate a deeper hue. Nonetheless, a minor decrease in L* was monitored with further raising the anthocyanin content between ACY_5_ and ACY_7_, which can be ascribed to a decreased rate of dye uptake (encapsulation). After exposure to food spoilage (30 h), L* considerably increased to indicate a color change from purplish to green. Under atmospheric conditions, increasing the anthocyanin quantity was shown to increase +a* and decrease –b* to indicate a purple color. After exposure to food spoilage (30 h), increases in increases in –a* and increases +b* were observed, indicating a greener color. Exposure to food spoilage (30 h) resulted in a color change from purplish to green, which was proved by a change from +a* to –a* values, and a change from –b* to +b* values.

**Figure 7:**
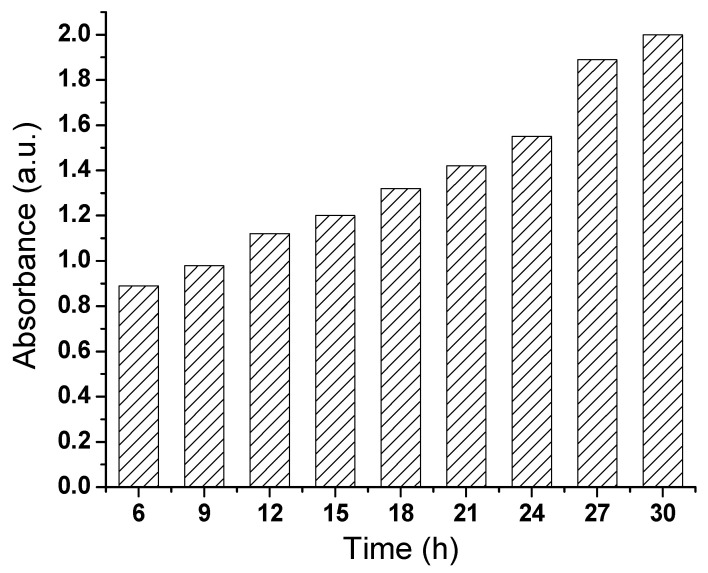
The calibration profile of ACY_5_ at 412 nm against the time period of exposure to rotten shrimp (6–30 h).

Using ACY_5_, a calibration curve was developed to monitor shrimp rotting at different periods ranging from 6 h to 30 h, as illustrated in Figure 7. The absorbance intensity demonstrated a nonlinear profile at 412 nm versus TVB-N accumulation. Applying a time exposure to food spoilage as low as 6 h, the absorbance wavelength shifted from 580 nm to 412 nm (an absorption intensity of 0.897). When the TVB-N content was accumulated over 30 h, the absorption intensity at 412 nm increased from 0.897 to 0.203, respectively.

**Figure 8:**
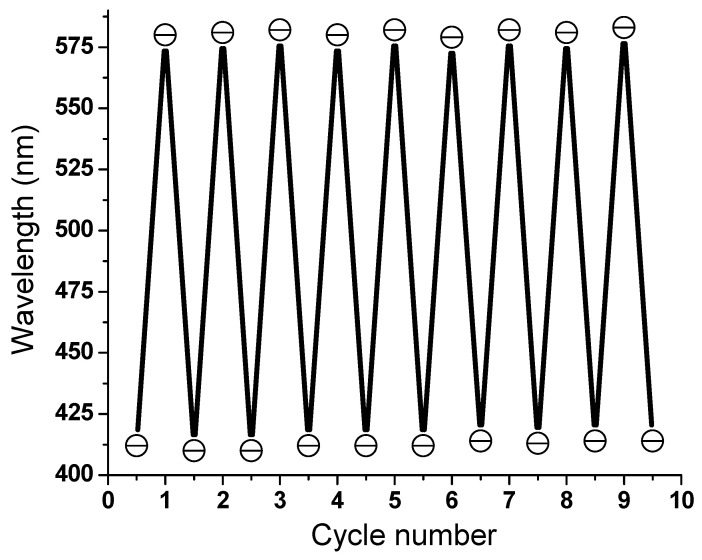
Reversibility of ACY_5_ over several cycles of exposure to rotten shrimp for 30 h (green, 412 nm) and air (purple, 580 nm).

In an experiment that tested the reversibility of the colored samples, the originally purple (580 nm) nanofibrous fabric (ACY_5_) was subjected to food spoilage, turning to green (412 nm). The nanofibrous cloth returned to its original purple color after a short time of exposure to atmospheric conditions. The abovementioned procedure was conducted over several cycles, while recording the highest absorbance wavelength. The results showed no change in the absorption spectra after a number of exposure cycles to rotten shrimp and air, proving strong reversibility without fatigue (Figure 8).

### 3.5. Proposed Mechanism

**Scheme 3:**
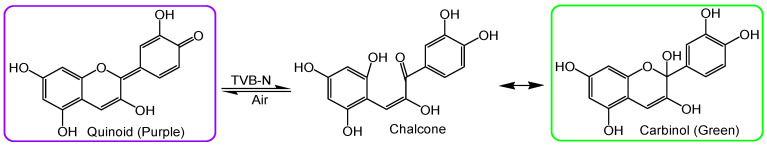
Proposed mechanism of TVB-N sensing demonstrating TVB-N-induced color shift of ACY-encapsulated cellulose nanocrystal-reinforced polyvinyl alcohol nanofibrous membrane from purple to green.

Anthocyanins are a flavonoid class of dyestuffs that can alter color by changing the pH value of the adjacent medium. They have shown a range of colors in aqueous solutions, including purple for a pH of 7, pink and red for a pH < 7, and colorless and greenish yellow at a pH > 7 [29,30,31,32,33]. As illustrated in Scheme 3, the monitoring of rotten shrimp was achieved by a change in color from purplish to green. Increasing the content of the alkaline TVB-Ns, such as ammonia and other nitrogen-based amines, leads to a noticeable increase in the pH value and deprotonation of anthocyanin. In order to monitor the shrimp deterioration, the sensor membrane (ACY_5_) was positioned in direct contact with shrimp to demonstrate a change in color from purplish to green. The anthocyanin probe has been used as a pH indicator. In addition, food spoilage, such as that of shrimp, generates TVB-N, which increases the pH of the medium [50]. Due to the accumulation of TVB-N, increasing the exposure duration to rotten shrimp resulted in a higher pH value [51]. As the total content of the basic TVB-N increased, a considerable increase to a higher pH value was monitored. Scheme 3 displays a change in color from purple to greenish in the presence of shrimp spoilage (TVB-N) due to TVB-N-driven deprotonation of anthocyanin. Numerous methods have been presented for food spoilage monitoring, such as chromatographic and amperometric techniques [52,53], and cellulose–naphthoquinone composites [54]. However, those detection methods are costly, not portable, time-consuming, or complicated. Additionally, the detection of food spoilage by colorimetric sensors has shown to be rapid, precise, cheap, and simple [55]. Herein, an efficient, selective, sensitive, cheap, biodegradable, and non-toxic sensor for food spoilage was developed from a combination of natural materials, including cellulose nanocrystal-reinforced polyvinyl alcohol nanofibrous membranes and anthocyanin biomolecules.

## 4. Conclusions

The current study reports the development of a reversible colorimetric nanofibrous sensor for food spoilage from a blend of natural materials, including cellulose nanocrystal-reinforced polyvinyl alcohol and anthocyanin biomolecule. Pomegranate extract was investigated by HPLC, indicating the presence of numerous phenols. TEM images of CNC indicated diameters of 12–17 nm. The nanoparticle diameter of M/ACY was reported in the range of 8–13 nm, and the diameter of the ACY/CNC@PVA nanofibers was in the range of 70–135 nm. The developed assay functions as a halochromic sensor, demonstrating a colorimetric change from purplish to greenish in response to the TVB-N released from food spoilage. When encapsulated as a direct dyestuff in cellulose nanocrystal-reinforced polyvinyl alcohol nanofibers in the existence of an alum mordant, the anthocyanidin extract from pomegranate (*Punica granatum* L.) generated nanoparticles of the M/ACY coordinating complex. After exposure to rotten shrimp, the CIE Lab coordinates and absorbance spectra proved a colorimetric shift from purple to green. Responsiveness to food spoilage was accomplished over a period of 6–30 h. The accumulation of TVB-N over time caused a blueshift from 580 nm to 412 nm. The stiffness, air permeability, and colorfastness were studied to determine the comfort properties of the membranes. The integration of M/ACY into the nanofibers had small effects on the air permeability and stiffness of the fibrous membranes. The present halochromic fabric can be reported as a simple, sensitive, biodegradable, and reversible colorimetric detector for food spoilage.

## Figures and Tables

**Table 1:** Phenols determined by HPLC in pomegranate extraction.

Phenol	Contents (%)
Gallic acid	1.80
Kaempferol	39.39
Catechin	1.84
Vanillin	1.43
Ferulic acid	9.24
Chlorogenic acid	1.36
Rutin	21.47
Caffeic acid	1.84
Acacetin	1.85
Cinnamic acid	3.75
Quercetin-7-methylether	1.94
Quercetin-7,3′-dimethylether	6.34
*P-Coumaric acid*	3.82
Chrysin	1.93

**Table 2:** Elemental investigation (wt%) of ACY_0_ and ACY_5_ at three sites (*St*_1_, *St*_2_, and *St*_3_).

Membrane		C	O	Al
ACY_0_		64.66	35.34	–
ACY_5_	*St*_1_	64.28	35.05	0.67
	*St*_2_	64.15	35.27	0.58
	*St*_3_	64.24	35.02	0.74

**Table 3:** Colorfastness screening of ACY-colored membranes.

Membrane	Washing		Perspiration				Light
	*St* *	*Alt* *	Acidic		Alkaline		
			*St*	*Alt*	*St*	*Alt*	
ACY_1_	4–5	4	4–5	4	4–5	4–5	7
ACY_2_	4	4	4	4	4	4	7
ACY_3_	4	4	4	4	4	4	7
ACY_4_	4	4	4	4	4	4	6–7
ACY_5_	4	4	4	4	4	4	6–7
ACY_6_	4	4	3–4	3–4	4	4	6
ACY_7_	3–4	3	3	3	3–4	3–4	5

* *Alt* is colorimetric alterations; *St* is cotton staining.

**Table 4:** Coloration screening of membranes before (*S*_1_) and after (*S*_2_) contact with rotten shrimp (30 h).

Sample	*K/S*		L*		a*		b*	
	*S*_1_	*S*_2_	*S*_1_	*S*_2_	*S*_1_	*S*_2_	*S*_1_	*S*_2_
ACY_0_	0.46	0.59	93.36	93.05	0.01	0.06	1.16	1.08
ACY_1_	1.63	0.89	52.15	73.32	4.20	−4.39	−31.27	5.66
ACY_2_	2.61	1.10	48.95	71.32	7.97	−7.04	−27.21	13.81
ACY_3_	3.73	1.22	45.21	66.99	12.56	−9.74	−22.16	21.07
ACY_4_	5.00	1.39	41.70	65.53	15.99	−10.58	−18.66	21.25
ACY_5_	6.32	1.57	39.86	64.37	18.02	−12.64	−16.75	22.90
ACY_6_	7.20	1.65	39.26	63.78	18.32	−13.79	−15.04	23.26
ACY_7_	7.46	1.83	38.94	63.52	18.61	−14.42	−14.23	23.75

## Data Availability

The data that support the findings of this study are available from the corresponding author upon reasonable request.

## Supplementary Materials

The following supporting information can be downloaded at: https://www.mdpi.com/article/10.3390/nano14211701/s1, Figure S1: Figure S1. FTIR spectrum of the ACY_0_ sample; Figure S2. FTIR spectrum of the ACY_1_ sample; Figure S3. FTIR spectrum of the ACY_7_ sample.
