# Supplementary material for: Fabrication of Anthocyanidin-Encapsulated Polyvinyl Alcohol Nanofibrous Membrane for Smart Packaging
Source: Nanomaterials (Basel). 2024 Oct 24;14(21):1701. doi: 10.3390/nano14211701 (PMC11547423; doi:10.3390/nano14211701)
Supplement: Supplementary file 1 [file nanomaterials-14-01701-s001.zip › nanomaterials-3243986-supplementary.pdf]

Supplementary Data

Fabrication of Anthocyanidin-Encapsulated Polyvinyl Alcohol Nanofibrous Membrane for Smart Packaging

Maryam Aldoghaim [1,*], Jabrah Alkorbi [2], Salhah D. Al-Qahtani [3] and Ghadah M. Al-Senani [3,*]

1 Department of Chemistry, College of Science, King Faisal University, Al-ahsa 31982, Saudi Arabia

2 Department of Chemistry, College of Science, The University of Sheffield, Sheffield S10 2TN, UK

3 Department of Chemistry, College of Science, Princess Nourah bint Abdulrahman University, P.O. Box 84428, Riyadh 11671, Saudi Arabia

* Correspondence: maldoghaim@kfu.edu.sa (M.A.); gmalsnany@pnu.edu.sa (G.M.A.-S.)

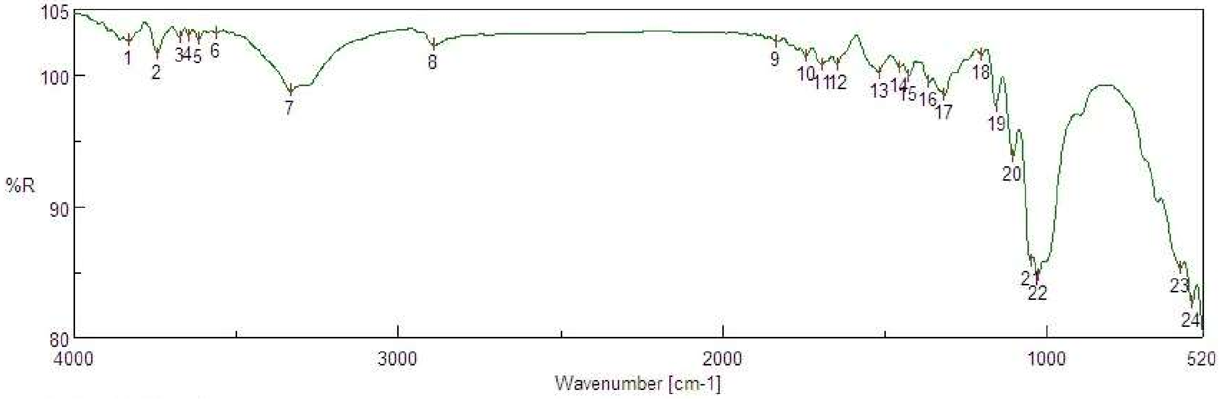

Figure S1. FTIR spectrum of the ACY_0 sample.

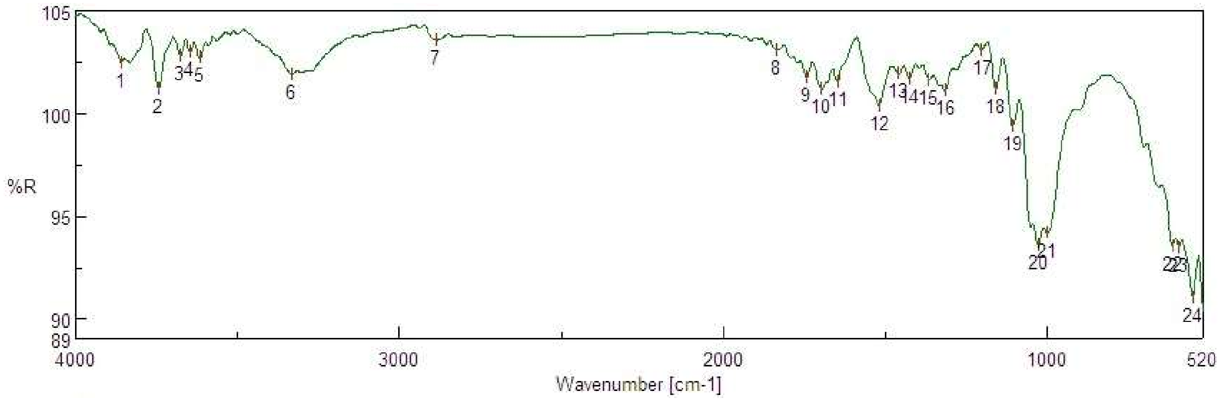

Figure S2. FTIR spectrum of the ACY_1 sample.

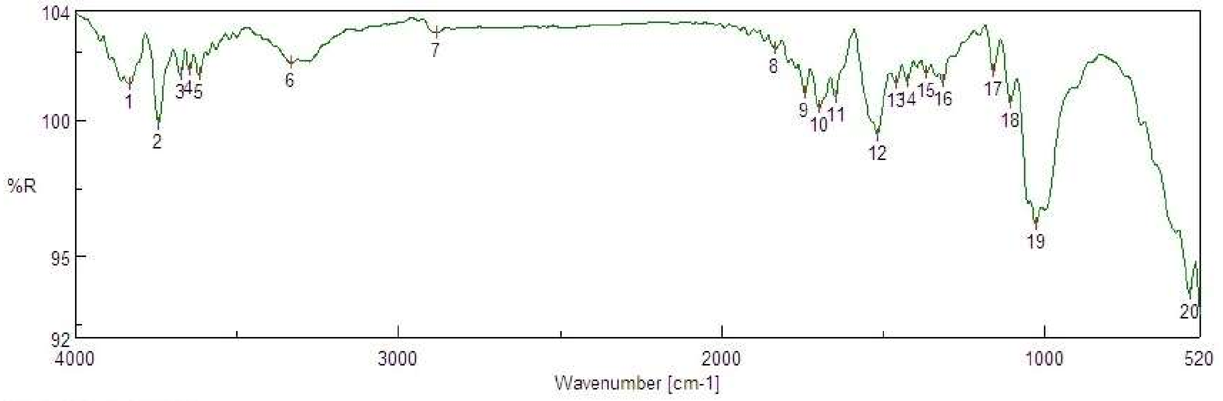

Figure S3. FTIR spectrum of the ACY_7 sample.
